# A novel PCR-based point-of-care method enables rapid, sensitive and reliable diagnosis of *Babesia gibsoni* infection in dogs

**DOI:** 10.1186/s12917-019-2181-5

**Published:** 2019-11-29

**Authors:** I-Li Liu, Nai-Yu Chi, Chia-Ling Chang, Ming-Long Hung, Chun-Ta Chiu, Hui-Wen Chen

**Affiliations:** 1 Institute of Veterinary Clinical Science, National Taiwan University, Taipei, Taiwan; 2 Credo Biomedical Pte. Ltd., Singapore, Singapore; 3 Department of Veterinary Medicine, National Taiwan University, 1 Sec 4 Roosevelt Rd, Taipei, Taiwan

**Keywords:** *Babesia gibsoni*, Point-of-care, QubeMDx PCR system, Real-time PCR

## Abstract

**Background:**

*Babesia gibsoni (B. gibsoni)* is an intraerythrocytic protozoan parasite of dogs that causes fever and hemolytic illness. A timely diagnosis is essential for the disease management.

**Results:**

Here, we report a QubeMDx PCR system which enables a rapid, sensitive and reliable diagnosis of *B. gibsoni* near the dog patient. Within 30 min, this diagnostic assay was able to detect as low as 0.002% parasitemia of the dog blood. Using clinical samples, this new assay was validated to demonstrate 100% agreement with real-time PCR.

**Conclusions:**

This novel diagnostic method provides a reliable point-of-care test to assist in the identification of *B. gibsoni*.

## Background

*Babesia gibsoni* (*B. gibsoni*) is a widespread tick-transmitted protozoan blood parasite that was first identified in 1910 in dogs and jackals from India and now prevalent in many regions, including Taiwan [1, 2] and other Asian countries such as Japan [3], Korea [4], China [5], Bangladesh [6], India [7] and Malaysia [8]. *B. gibsoni* infection is frequently found in companion dogs and presents a serious clinical problem in the world. The clinical severity of *B. gibsoni* infection in dogs is age-dependent and potentially life-threatening with clinical symptoms ranging from remittent fever and severe hemolytic illness. Therefore, the diagnosis of this disease and the detection of dogs that carry this blood parasite are very important [9–12].

Generally, the standard method for definitive means of diagnosis of babesiosis is microscopic examination of blood smears following Giemsa-staining for visual identification of parasites. Although this microscopic method is an inexpensive diagnostic test, the detection of *B. gibsoni* in erythrocytes on blood smears is sometimes difficult and may be error-prone. As *B. gibsoni* is a small and pleomorphic organism (1–3 μm in diameter), unlike the large, paired and teardrop-shaped *Babesia canis*, a highly experienced diagnostic skill is required to distinguish it from artifacts under the microscope, and it becomes even more challenging to find the parasites when an infected dog has low levels of parasitemia [12]. On the other hand, the molecular diagnosis detecting the DNA of *B. gibsoni* in canine blood samples by polymerase chain reaction (PCR) offers high degrees of detection sensitivity and specificity. Various conventional [13], nested [14] and real-time [15] PCR assays detecting *B. gibsoni* infection in dogs have been developed. However, these assays mostly require a relatively expensive thermo cycler and well-trained personnel, which may delay results and add costs. As a result, a rapid, affordable and user-friendly platform is needed for a point-of-care (POC) detection of *B. gibsoni* in daily veterinary practice to facilitate animal care and disease management.

The recently developed QubeMDx (Credo Biomedical Pte. Ltd., Singapore) is a portable diagnostic system (12.1 × 10.9 × 14.7 cm, H × W × D) using reaction tubes prefilled with fluorescence-based reagents to facilitate the real-time PCR detection process within 15 min. The programmed device allows users to perform four different targeted DNA or RNA molecular diagnosis in a given round of reaction with minimal training. The purpose of the present study is to evaluate the QubeMDx PCR system as a practical and accurate method for the diagnosis of *B. gibsoni* infection in dog by using clinical canine blood samples and comparing to a standard real-time PCR assay.

## Results

**Fig. 1:**
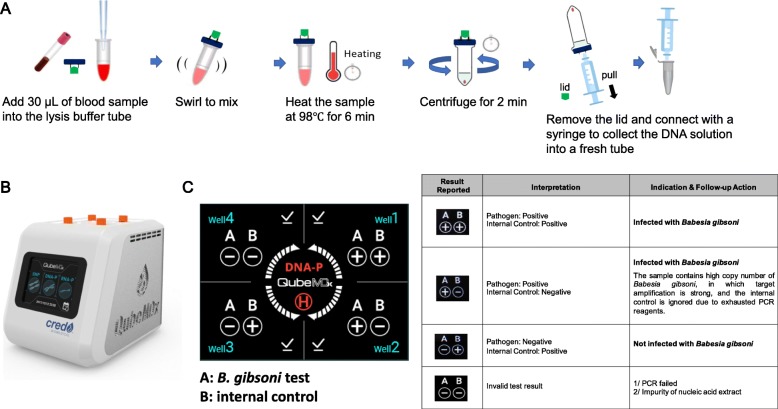
QubeMDx PCR assay for *B. gibsoni*. **a** Graphical illustration of the DNA extraction procedures using the Credo Rapid Blood Nucleic Acid Extraction Kit. **b** A photo of the QubeMDx PCR device. **c** The reading interface and interpretations of the QubeMDx PCR device. The test result for *B. gibsoni* is displayed in the panel A, either positive (+) or negative (−) on the screen, and the test validity (internal control) is displayed in the panel B

**Fig. 2:**
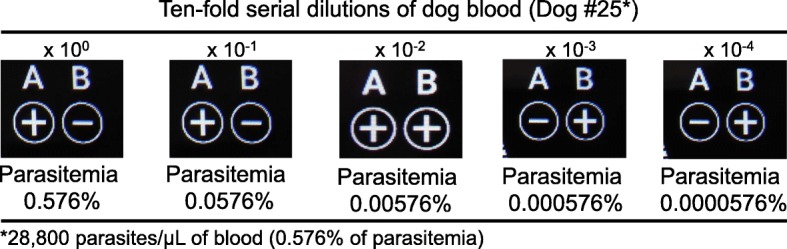
Detection limit of QubeMDx PCR assay for *B. gibsoni*. *B. gibsosi*-positive dog blood (0.576% of parasitemia according to the real-time PCR) was 10-fold serially diluted and tested onto the QubeMDx PCR system to determine the detection limit*.* The test result for *B. gibsoni* is displayed in the panel A, with positive (+) or negative (−) result. The test validity (internal control) is displayed in the panel B

As illustrated in Fig. 1a, the DNA extraction can be completed within 8 min. The resultant DNA solution was subsequently assayed using the QubeMDx PCR device (Fig. 1b). After 15-min reaction time, the test result for *B. gibsoni* was displayed in the panel A, either positive (+) or negative (−) on the screen, and the test validity (internal control) was displayed in the panel B (Fig. 1c). Evaluated using 28 reference test (real-time PCR)-positive clinical samples, the sensitivity of the QubeMDx PCR was 100%. The specificity was also 100%, calculated using 27 reference test-negative samples. The *Kappa* value was 1, indicating highly reproducible results between these two methods. Furthermore, among different levels of parasitemia analyzed from 28 positive dog samples (Table 1), the QubeMDx PCR system successfully detected the *B. gibsoni* ranging from 94 to 398,000 parasites/μL of blood (0.002 to 8% of parasitemia). Importantly, no discrepancy between these two methods was found among these clinical samples containing low levels of parasitemia (16/28) (< 5000 parasites/μL of blood or parasitemia < 0.1%). Using ten-fold serially diluted blood sample (dog #25), the QubeMDx PCR was shown to be able to detect 0.00576%, but not 0.000576% of parasitemia (Fig. 2). Collectively, DNA rapid extraction and QubeMDx PCR allow for detection of the *B. gibsoni* DNA with a sensitivity limit in between 0.000576 and 0.002% of parasitemia. The analytical specificity of the QubeMDx PCR for *B. gibsoni* detection was further assessed using blood samples positive for *Anaplasma platys, Ehrlichia canis* or *Babesia canis.* As indicated in Additional file 1: Table S1, no false positive detection results were obtained from canine blood samples infected with other three pathogens.

**Table 1:** *B. gibsoni*-positive dog blood samples included in this study

No.	Age (yr)	Sex	Breed	PCV^a^ (%)	Test results		Parasites/μL of blood^b^
					QubeMDx	Real-time PCR	
1	8	M	Mixed	n/a	+	+	94
2	7	M	n/a	32.3	+	+	98
3	12	F	Mixed	28.8	+	+	103
4	13	M	Mixed	21.3	+	+	188
5	4.3	F	Mixed	50.5	+	+	249
6	1.7	M	Miniature Schnauzer	23.6	+	+	261
7	14	F	Mixed	27.3	+	+	422
8	9	F	Mixed	31.8	+	+	798
9	2	F	Mixed	n/a	+	+	1030
10	5	M	Poodle	37.8	+	+	1150
11	8	M	Mixed	48.7	+	+	1290
12	6	F	Mixed	n/a	+	+	1360
13	5	M	Poodle	37.7	+	+	1870
14	6	F	Mixed	n/a	+	+	2180
15	5	F	Mixed	n/a	+	+	3610
16	9	M	Labrador Retriever	25.9	+	+	3840
17	4	F	Mixed	43.9	+	+	5410
18	6	M	Poodle	47.3	+	+	8140
19	3	M	Poodle	14.6	+	+	8140
20	2	F	Mixed	51.2	+	+	8760
21	0.2	F	Mixed	30.3	+	+	10,100
22	12	F	Miniature Schnauzer	16.7	+	+	10,800
23	9	F	Mixed	n/a	+	+	13,700
24	6	M	Corgi	10.5	+	+	14,200
25	2	M	Pomeranian	40.6	+	+	28,800
26	12	M	Dachshund	16	+	+	30,400
27	0.5	M	Bichon Frisé	18.6	+	+	149,000
28	10	M	Maltese	14.6	+	+	398,000

^a^Packed cell volume

^b^Parasites was determined by real-time PCR and calculated from the standard curve constructed with the plasmid DNA according to Matsuu et al., 2005 [15]

## Discussion

In this study, a novel and reliable POC diagnostic method for *B. gibsoni* detection was reported. By coupling the Credo Rapid Blood Nucleic Acid Extraction Kit and the QubeMDx PCR system, the entire diagnostic procedures for *B. gibsoni* can be completed within 30 min in clinic, where veterinarians primarily rely on microscopic examination as the diagnosis method. The simplified assay procedures and saved time from laboratory identification may expedite the prescription medications and lead to effective disease management for the affected dogs.

The detection limit of diagnostic tests is a critical parameter for accurate and timely pathogen identification. It has been suggested that *B. gibsoni* infection with low parasitemia (0–0.75%) is easily misdiagnosed as immune-mediated hemolytic anemia and sometimes inappropriately treated with immune suppressive drugs [16, 17]. For dogs with babesiosis, either during the early or chronic stages of infection or under medical treatment, the parasite numbers in the blood may be low. Previous experimental infection study has shown that, not until 10 days after infection, dogs inoculated with *B. gibsoni* revealed a significantly decreased level of packed cell volume (PCV) (< 37%) or with a parasitemia greater than 0.1% [15]. By using this sensitive QubeMDx PCR system, the detection limit of 0.002% of parasitemia (94 parasites/μL of blood) allows for early diagnosis of infected dogs. Although greater detection limit was previously demonstrated by the real-time PCR (9 parasites/μL of blood) [15], recombinase polymerase amplification-lateral flow (LF-RPA) dipstick method (0.5 parasites/μL of blood) [5], loop-mediated isothermal amplification (LAMP)(0.0000135% of parasitemia) [18], the differences can be attributed to the use of rapid DNA extraction procedures in the QubeMDx system, leading to a compromised DNA yield.

In addition to a low detection limit, a 100% of sensitivity and specificity for *B. gibsoni* detection was achieved by this QubeMDx PCR based on real-time PCR confirmed positive/negative samples, while previously 86.67 and 57.33% of positive rate were obtained by the LF-RPA dipstick method [5] and the LAMP method [18], respectively, evaluated from clinically suspected dogs. Furthermore, the internal control included in each reaction of QubeMDx PCR provides an additional source for quality control. Taken together, compared to other available molecular methods, QubeMDx PCR system is a reliable POC method with advantages of rapidity and reaction simplicity.

## Conclusions

This reported QubeMDx PCR system offers a rapid (< 30 min), sensitive, and effective diagnosis for *B. gibsoni* in clinical infections. The present point-of-care testing allows for treatment prescription in the early stages of the infection, facilitating the recovery of affected dogs.

## Methods

A total of 57 EDTA-anticoagulated canine whole blood samples were collected at the National Taiwan University Veterinary Hospital. For the reference test of *B. gibsoni* detection, total DNA was extracted from 100 μL of blood sample with the DNeasy Blood & Tissue Kit (Qiagen, Hilden, Germany) according to the manufacturer’s instructions. A real-time PCR for *B. gibsoni* detection was performed to define the infection status and the standard curve was constructed with plasmid DNA for the conversion of parasitemia [15]. Through the reference test, 28 samples were defined as positive and used for the sensitivity evaluation for QubeMDx PCR system, whereas other 29 samples were negative and used for the specificity determination. In addition, the detection limit of the QubeMDx PCR system was determined using serially-diluted dog blood from one of the real-time PCR-positive blood sample. Furthermore, six canine whole blood samples diagnosed as *Ehrlichia canis*, *Anaplasma platys* or *Babesia canis* positive by PCR or real-time PCR [19–21] were also included in this study to evaluate the specificity of the QubeMDx PCR system.

To conduct the QubeMDx PCR assay, DNA was prepared by using the Credo Rapid Blood Nucleic Acid Extraction Kit (Credo Biomedical Pte. Ltd.). Briefly, 30 μL of blood sample was added to the tube containing the lysis buffer and the mixture was heated at 98 °C for 6 min. After the heating, the reaction mix was fractionated via centrifugation, and total DNA in the supernatant was collected to a fresh tube. The resultant DNA solution was subsequently assayed using the QubeMDx *Babesia gibsoni* Detection Kit (Credo Biomedical Pte. Ltd.) according to the manufacturer’s instructions. Briefly, 30 μL of the DNA solution was added to the reaction tube prefilled with lyophilized reagents containing primers and fluorogenic probes targeting the p18 gene of *B. gibsoni* as well as PCR essential reagents and internal controls. Tubes were then loaded onto the QubeMDx PCR device, and the fluorescent signals during amplification were detected and processed by a built-in algorithm. The video demonstrating assay procedures was filmed and can be found in the following link: https://www.dropbox.com/s/nbdt9ryvuubxsea/QubeMDx.mp4?dl=0.

## Supplementary information

**Additional file 1: Table S1.** Analytical specificity results of the QubeMDx PCR for *B. gibsoni*.

## Data Availability

The data used and/or analyzed during the current study are available from the corresponding author on reasonable request.

## Abbreviations

*B. gibsoni*: *Babesia gibsoni*
LAMP: Loop-mediated isothermal amplification
LF-RPA: Recombinase polymerase amplification-lateral flow
PCR: Polymerase chain reaction
PCV: Packed cell volume
POC: Point-of-care
