# Supplementary material for: A novel PCR-based point-of-care method enables rapid, sensitive and reliable diagnosis of Babesia gibsoni infection in dogs
Source: BMC Vet Res. 2019 Nov 29;15:428. doi: 10.1186/s12917-019-2181-5 (PMC6884907; doi:10.1186/s12917-019-2181-5)
Supplement: Supplementary file 1 — Additional file 1: Table S1. Analytical specificity results of the QubeMDx PCR for B. gibsoni. [file 12917_2019_2181_MOESM1_ESM.pptx]

## Slide 1
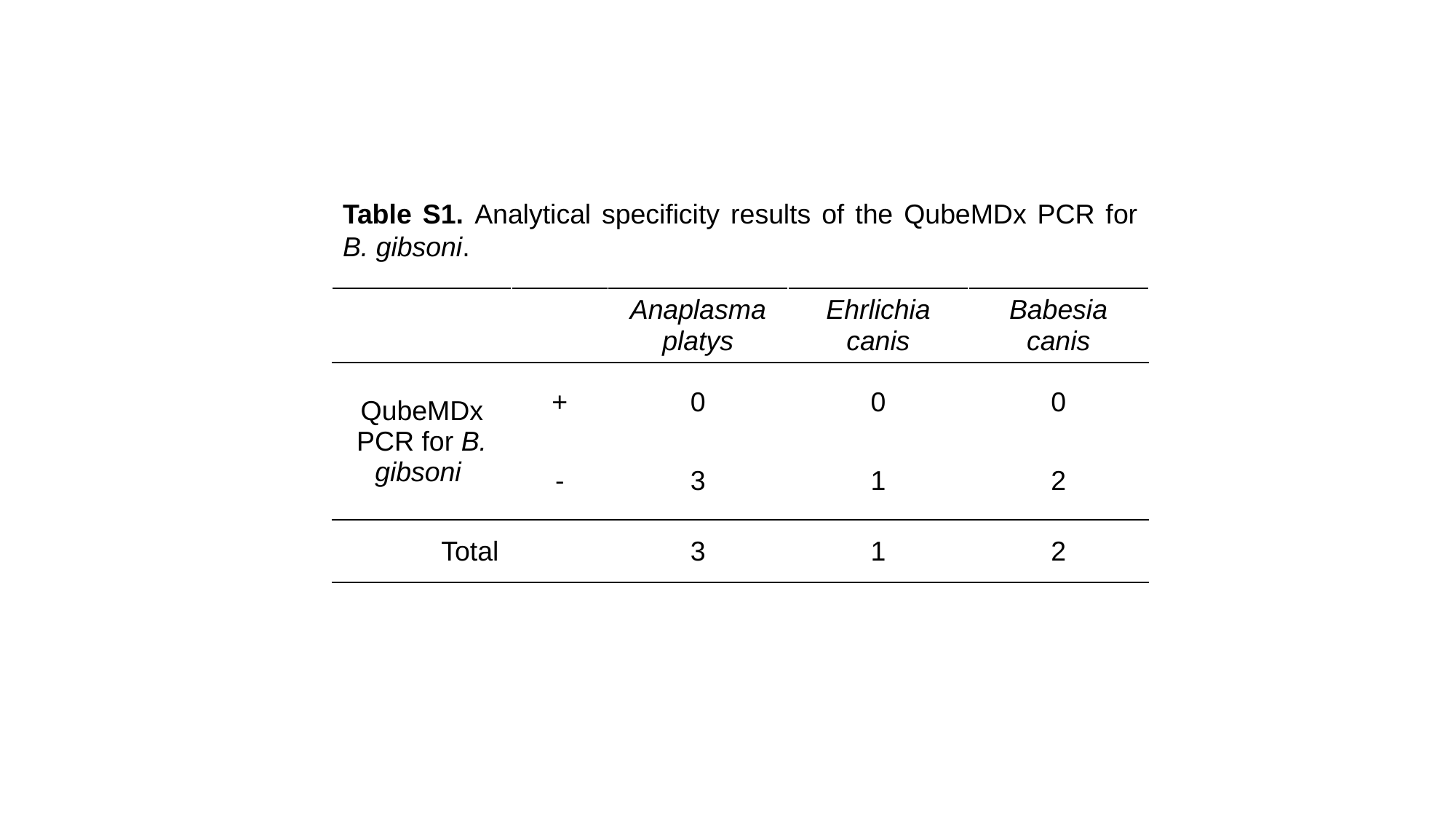

Table S1. Analytical specificity results of the QubeMDx PCR for B. gibsoni.
| | | Anaplasma platys | Ehrlichia canis | Babesia canis |
| --- | --- | --- | --- | --- |
| QubeMDx PCR for B. gibsoni | + | 0 | 0 | 0 |
| | - | 3 | 1 | 2 |
| Total | | 3 | 1 | 2 |

## Slide 2
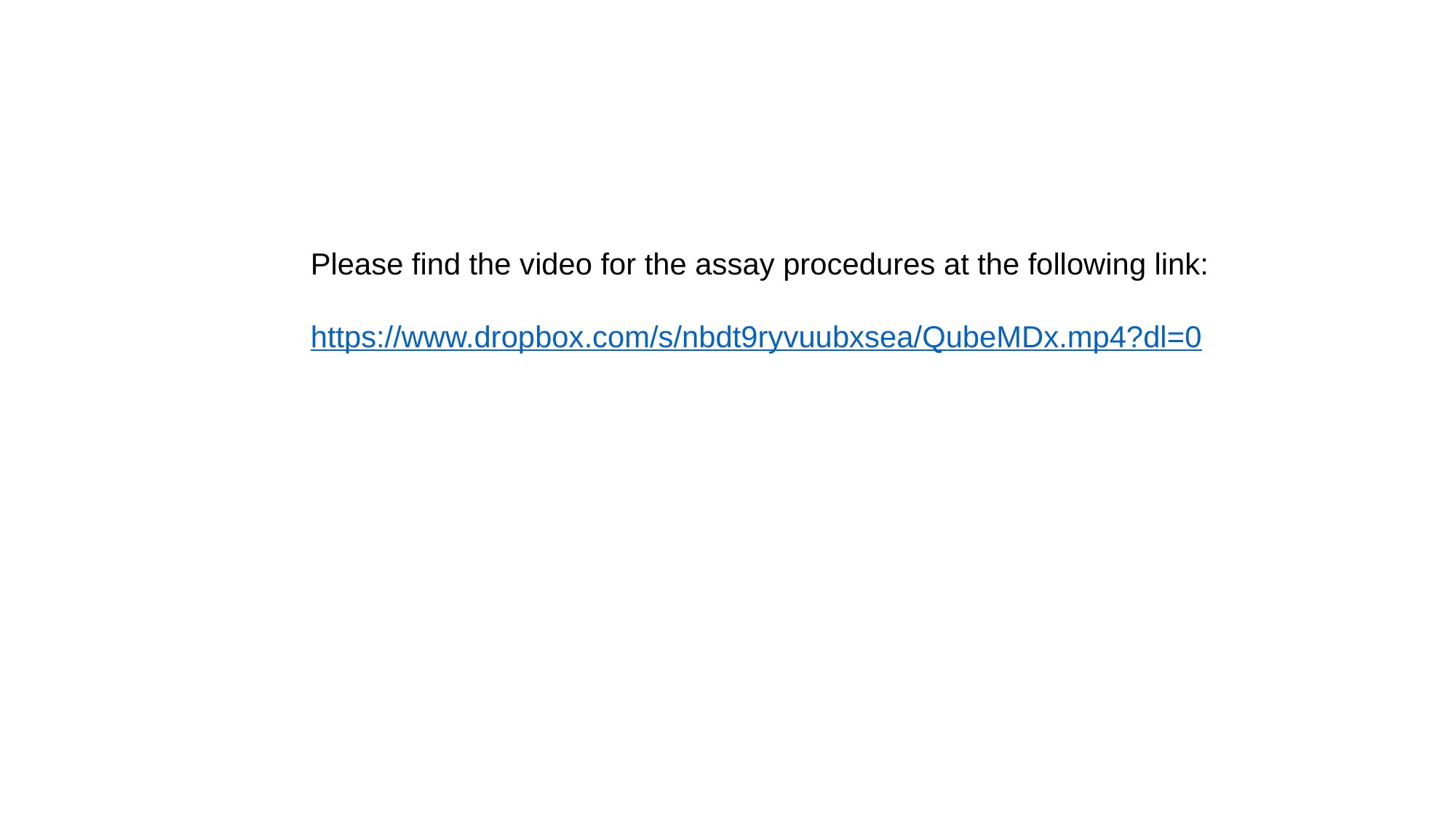

Please find the video for the assay procedures at the following link:
https://www.dropbox.com/s/nbdt9ryvuubxsea/QubeMDx.mp4?dl=0
